# Prediabetes as a risk factor for stress induced hyperglycemia in non diabetic critically ill adult

**DOI:** 10.1186/2197-425X-3-S1-A187

**Published:** 2015-10-01

**Authors:** DDJ Garcia-Gallegos, E Gomez-Sandoval, MN Gomez-Gonzalez, R Soriano-Orozco, E Luis-Lopez, MDR Valdez-Medina

**Affiliations:** University of Guanajuato, Medicine, Guanajuato, Mexico

## Introduction

Hyperglycemia in critically ill patients has not shown predict poor outcomes in those with preexisting Diabetes Mellitus (DM). Otherwise hyperglycemia in patients without DM has shown poor outcomes and generally this association reflects the severity of the disease. In addition the high level of glycemia can produce deleterious effects and is associated inversely to the survival. Also, It has been shown that insulin has anti-inflammatory effects, and hyperinsulinism can predicts survival in experimental critically ill models. Nevertheless is not known if prediabetic patients (A subgroup of non-diabetic patients that often presents insulin resistance and hyperinsulinism) have an increased risk of stress induced hyperglycemia.

## Objectives

The goal of this study is to determine if prediabetes is a risk factor for stress induced hyperglycemia in critically ill adult.

## Methods

A descriptive, observational, transversal study was conducted to obtain the risk of prediabetic patient to present stress induced hyperglycemia. Patients without history of prediabetes were recruited in a period of one month and obtained the glycosylated hemoglobin and classified as diabetic and non-diabetic. Those with DM (glycosylated hemoglobin >6.5%) were excluded, and those without DM were classified as prediabetic or not, including patient with prediabetes in the range of glycosylated hemoglobin from 5.7 to 6.4%. We considered stress induced hyperglycemia a central glucose more than 140 mg/dl at admission. We did the screening of 15 patients; 2 of them (13.3%) were excluded because a glycosylated hemoglobin more than 6.5%, the other 13 patient (86.7%) were included in the study. With this information we obtained relative risk (RR), fraction and risk attributable to exposure and its significance.

## Results

**Figure 1:**
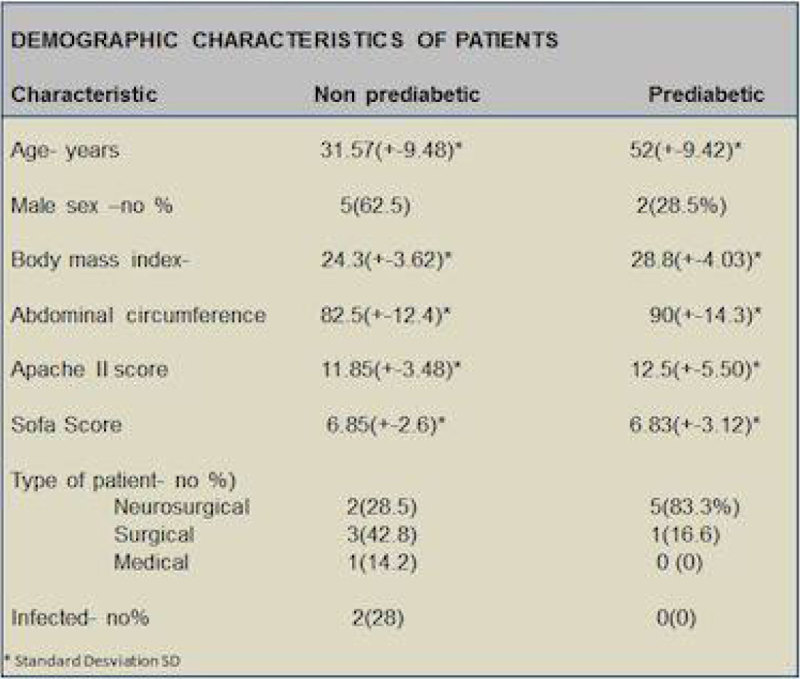
**Demographic chart.**

We obtain 6 patients (40%) from our total screening in the range of prediabetes. From those with prediabetes 5 of the 6 patients (83.3%) developed stress induced hyperglycemia, and those without prediabetes only 1 of 7 (14.2%). We obtain a relative risk (RR) of 5.86, IC95%(1.09,36.89) p < 0.05, fraction and risk attributable to exposure were 82.9% and 69% respectively. Figure 1.

## Conclusions

In critically ill non diabetic adult, prediabetes increases 5.86 times the risk of stress induced hyperglycemia. More studies are needed to determine if hyperglycemia in this group of patient affects ICU stay, morbidity and mortality.

## Grant Acknowledgment

This study did not receive any grant from any funding agency.
